# Supplementary figures and images for: Drug dosage modifications in 24 million in-patient prescriptions covering eight years: A Danish population-wide study of polypharmacy
Source: PLOS Digit Health. 2023 Sep 7;2(9):e0000336. doi: 10.1371/journal.pdig.0000336 (PMC10484442; doi:10.1371/journal.pdig.0000336)

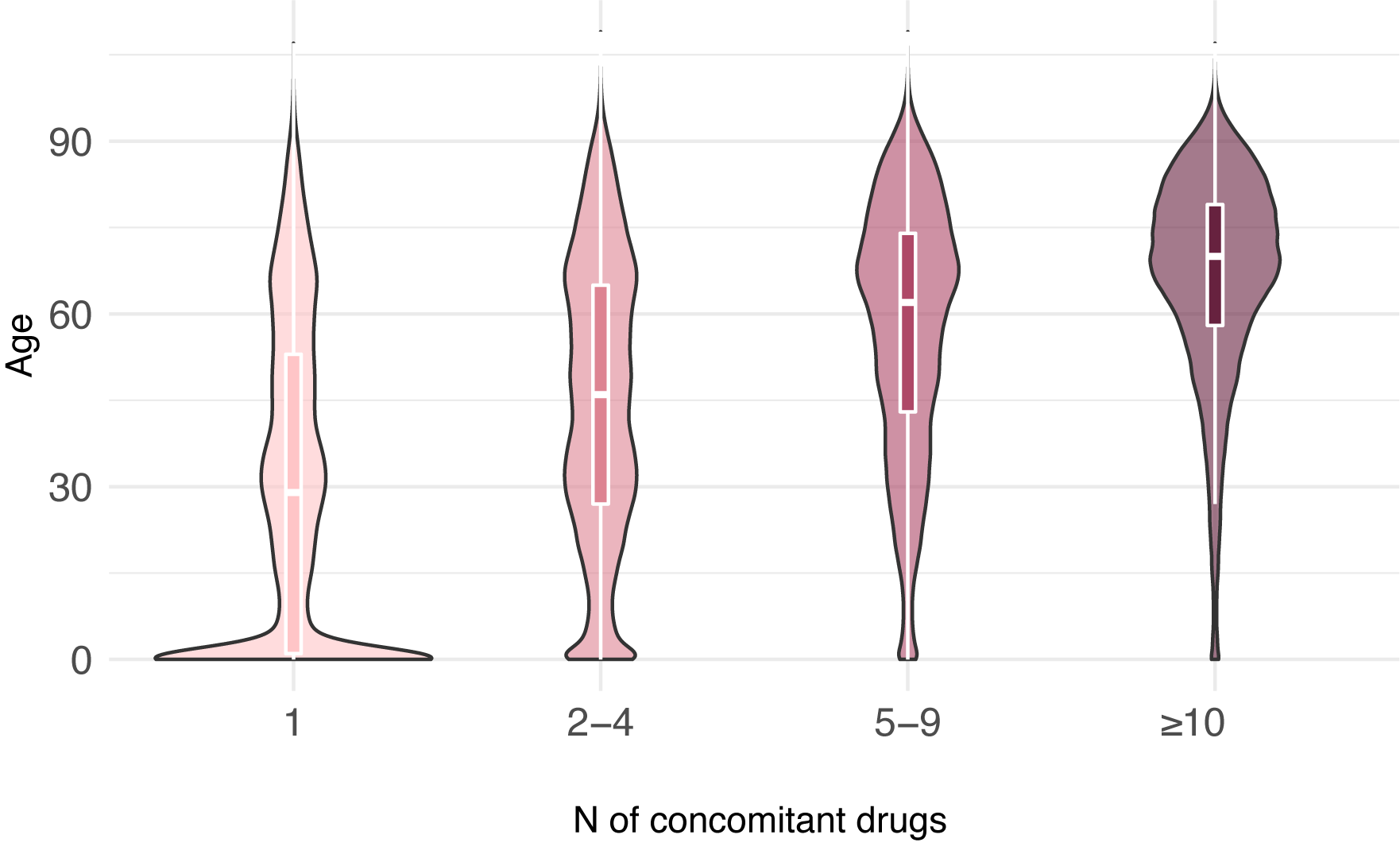

Supplement: S1 Fig — Age distribution based on the number of concomitant drugs (1, 2–4, 5–9, ≥10). Pearson ρ:0.40, 95% confidence interval CI:0.39–0.40, p-value < 2.2x10^-16^. CI: Confidence Interval. (TIF) [file pdig.0000336.s001.tif]

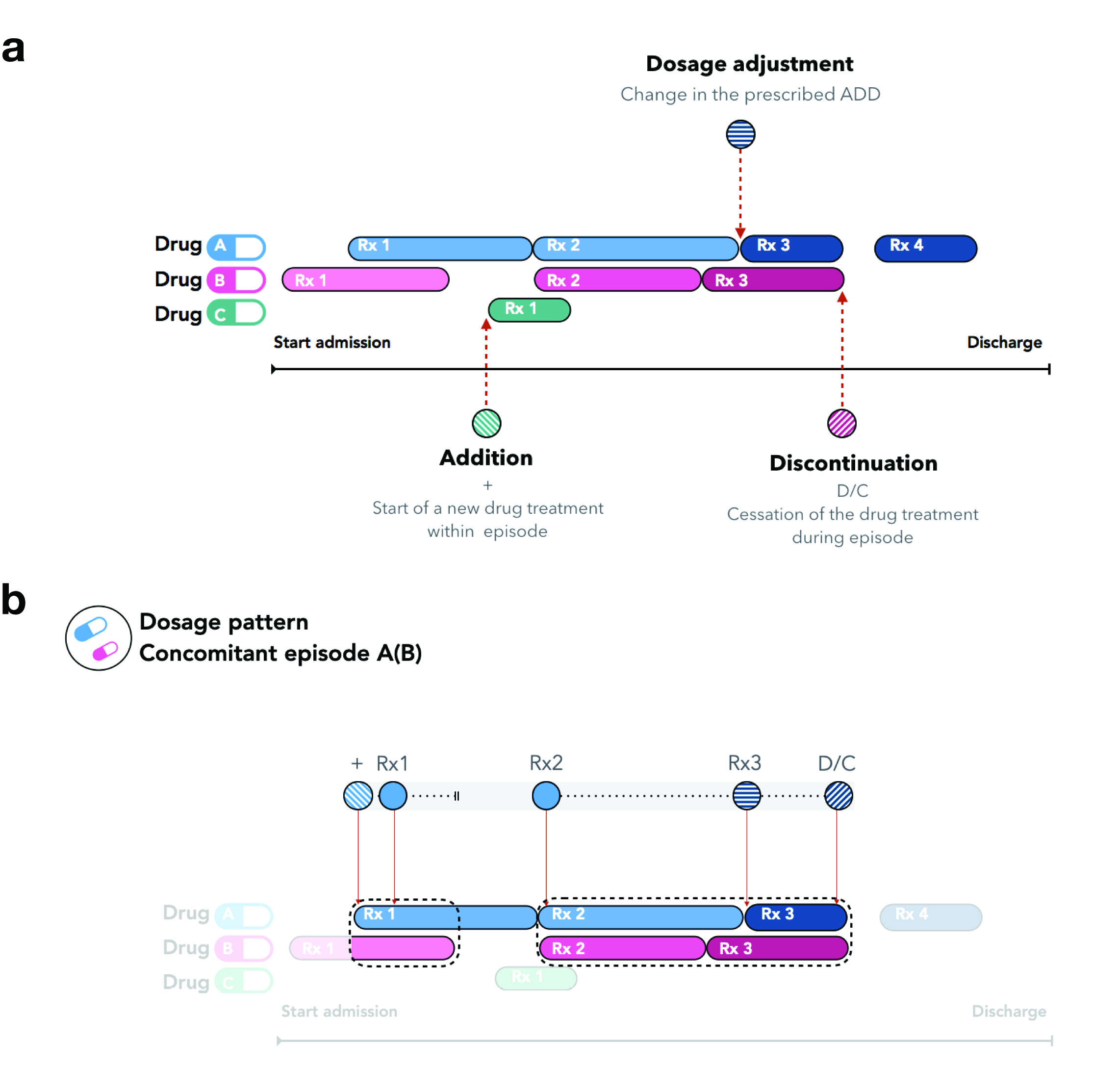

Supplement: S2 Fig — a Example of an inpatient admission where three index drugs (drug A, drug B and drug C) are administered. Timestamped prescriptions (Rx n) are shown for each drug (four for drug A, three for drug B and one for drug C), the colour grading indicates the prescribed average daily dose (ADD). b For each index drug, concomitant treatment episodes are defined as the co-medication overlap between a pair of drugs. The dotted area exemplifies the concomitant episodes for the index drug A with co-medication drug B, A(B). During A(B), the index drug A is added, followed by three consecutive prescriptions (Rx1, Rx2, Rx3) and then discontinued (D/C). (TIF) [file pdig.0000336.s002.tif]

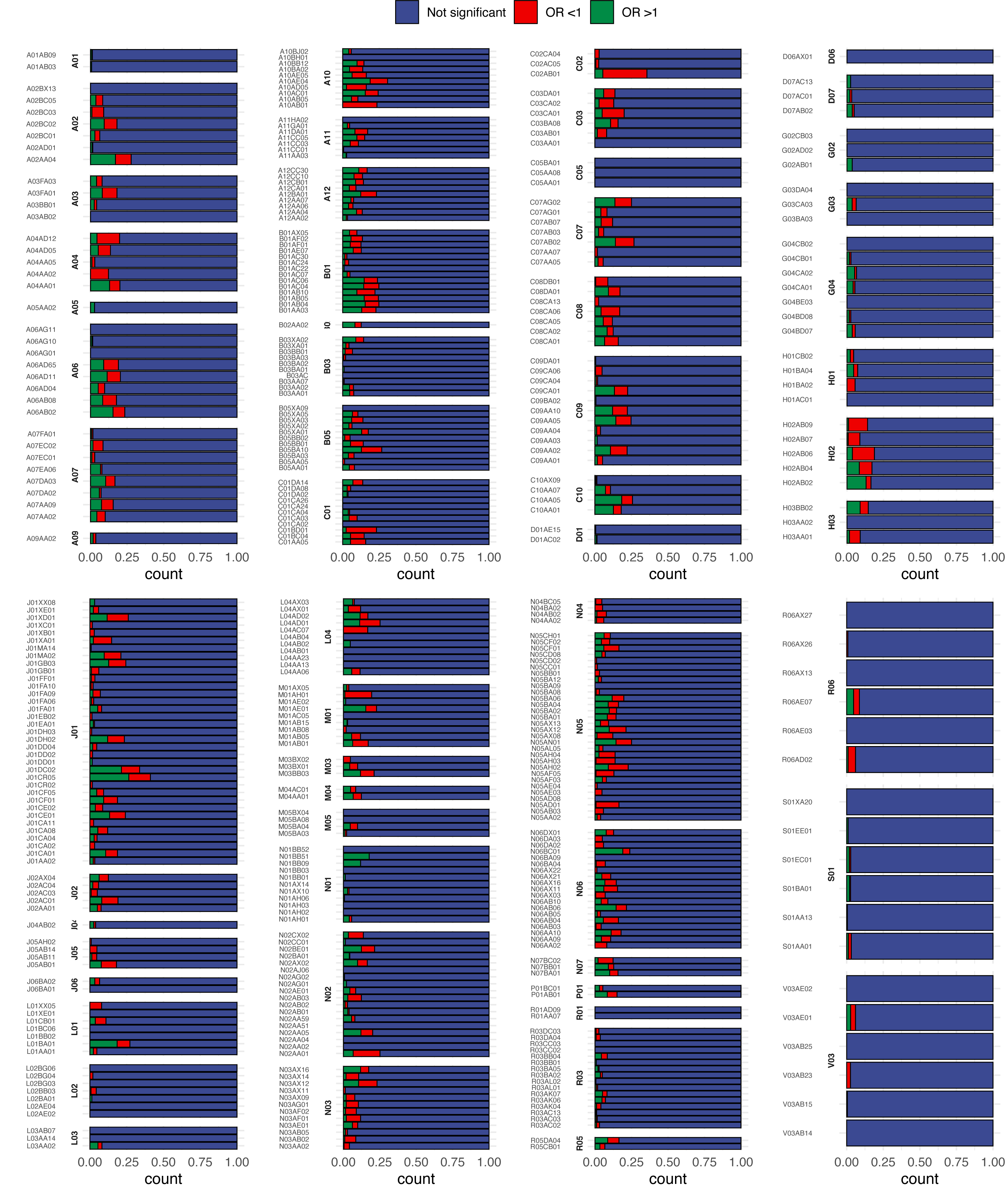

Supplement: S3 Fig — Stacked barplots representing the proportion of co-medications correlated with co-medications for each index drug. The index drugs with the highest proportion of co-medications with ORs >1 were: piperacillin (J01CR05), cefuroxime (J01DC02), caffeine (N06BC01), insulin glargine (A10AE04), methotrexate (L01BA01) and atorvastatin (C10AA05), consisting of more than 18% of all their co-medications. OR: Odds Ratio. (TIF) [file pdig.0000336.s003.tif]

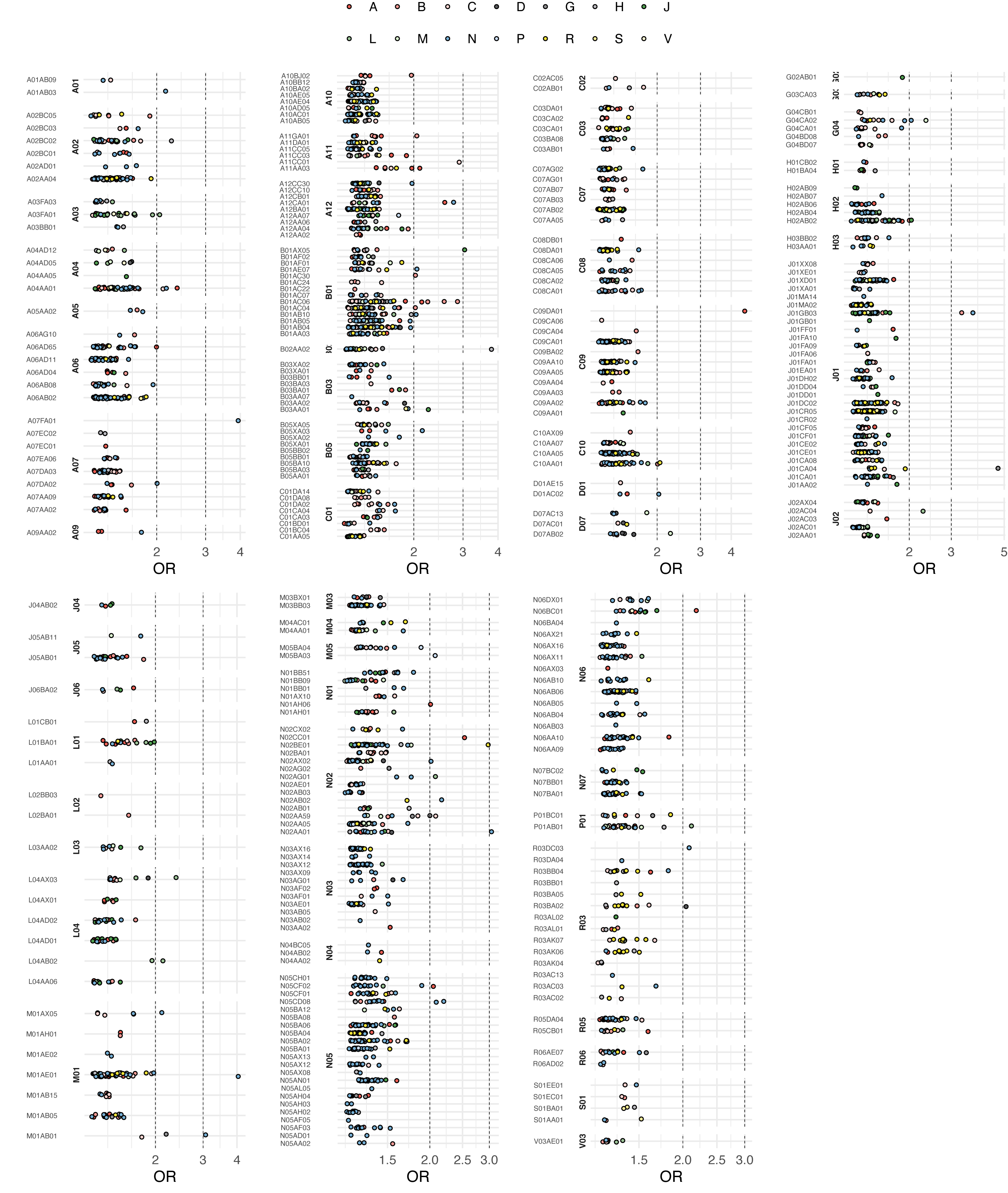

Supplement: S4 Fig — Representation of the 309 index drugs with dosage adjustments having ORs >1 with co-medications. Each point represents the OR of a co-medication for the given index drug. OR: Odds Ratio. (TIF) [file pdig.0000336.s004.tif]

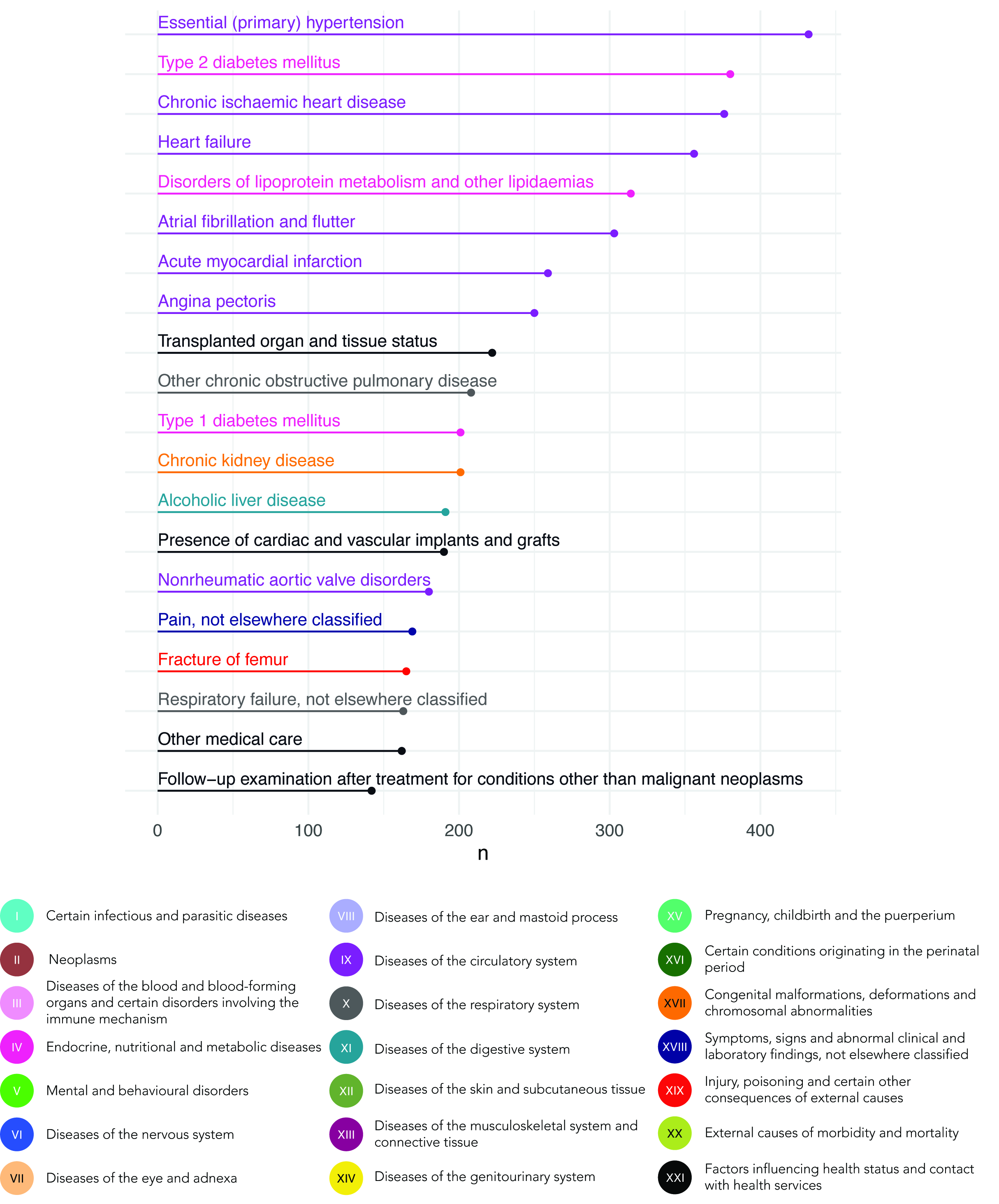

Supplement: S5 Fig — Segment bars indicate the number (n) of co-medication pairs positively correlated with each diagnosis over the total 3,993 dosage adjusted co-medication pairs. Colour of the bars correspond to the chapter level of the ICD-10 classification of diseases. (TIF) [file pdig.0000336.s005.tif]

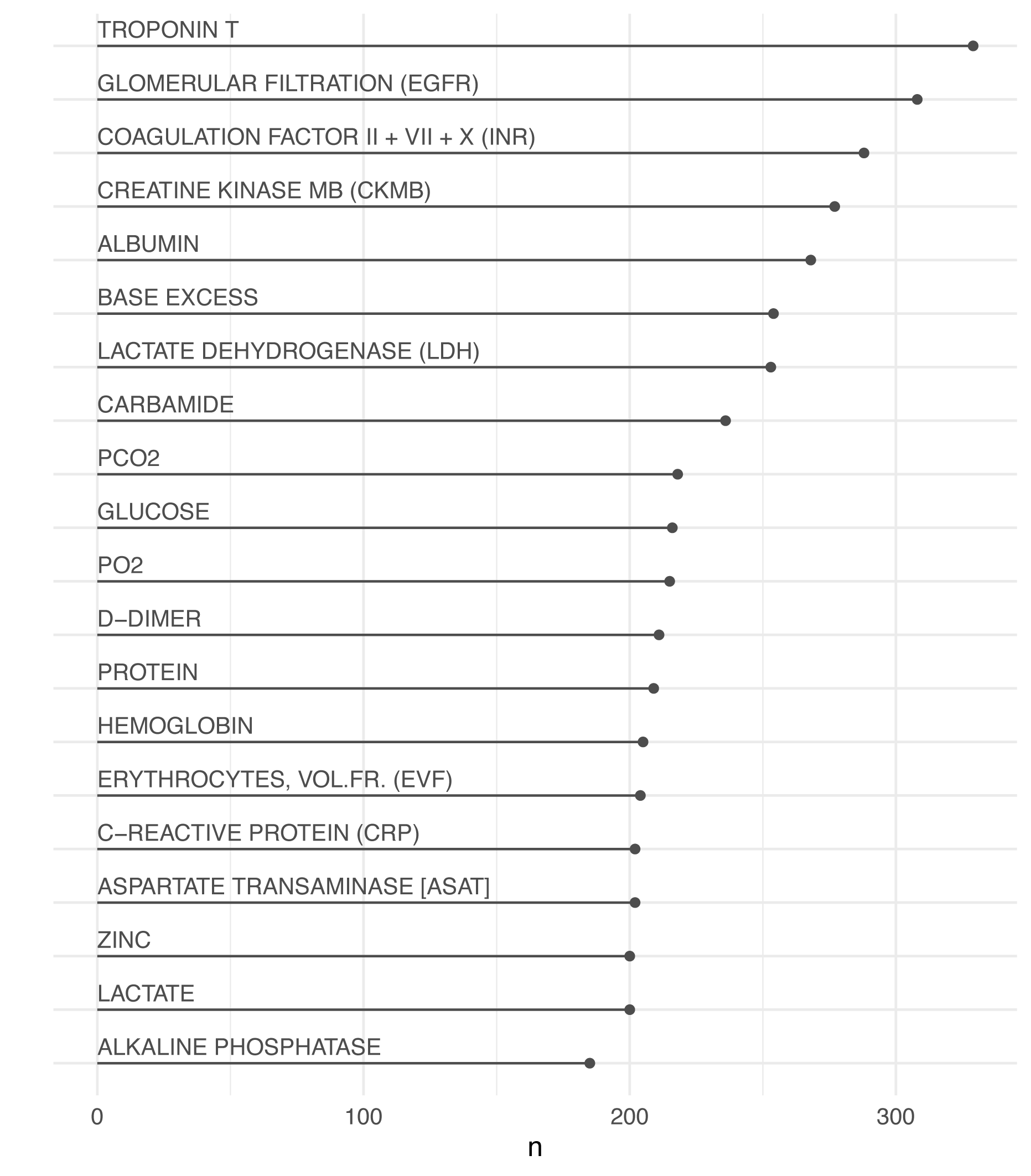

Supplement: S6 Fig — Segment bars indicate the number (n) of co-medication pairs positively correlated with each laboratory test over the total 3,993 dosage adjusted co-medication pairs. (TIF) [file pdig.0000336.s006.tif]

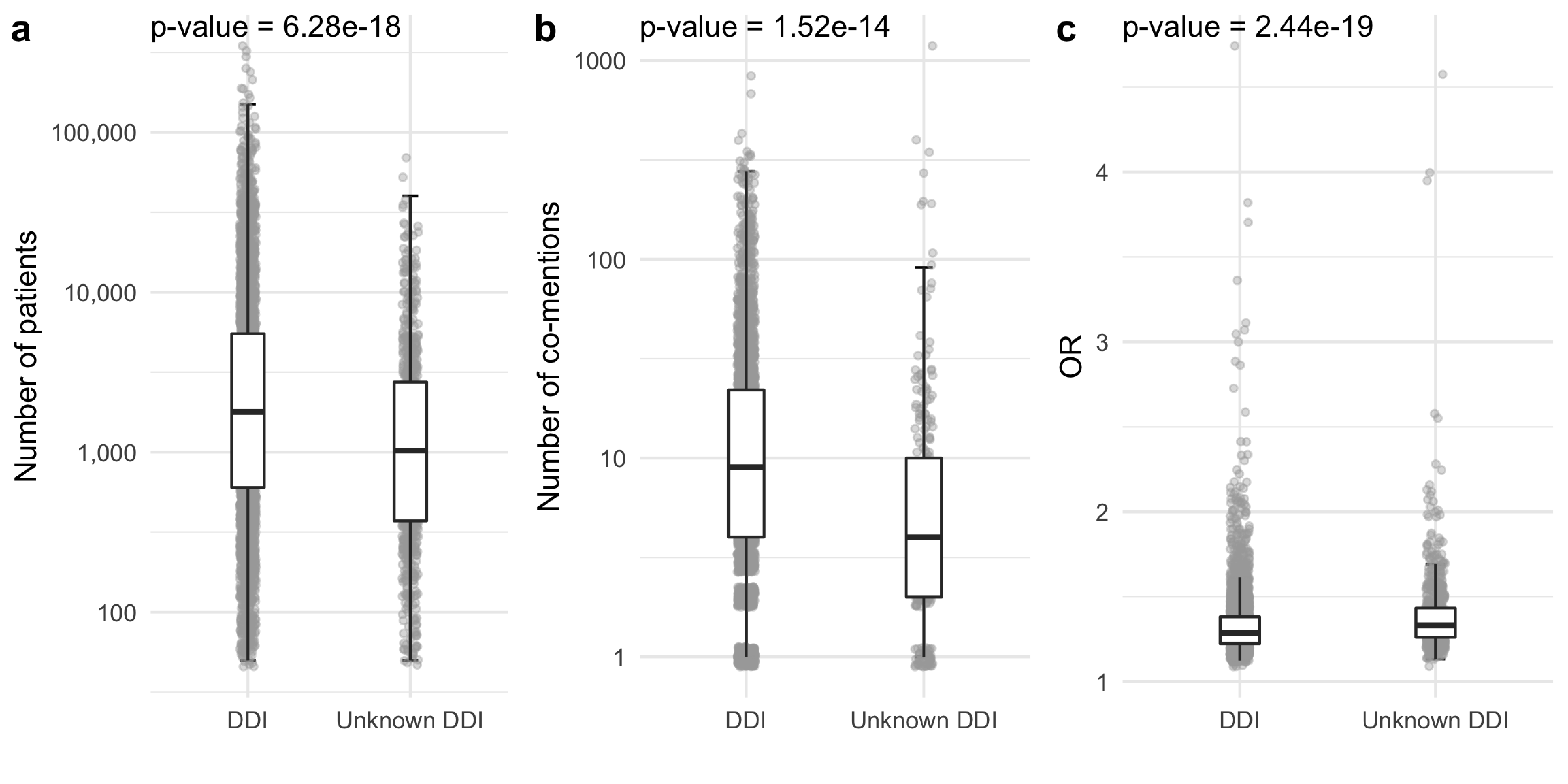

Supplement: S7 Fig — a Boxplots indicating the number of patients and the DDI evidence among co-medication pairs with ORs >1 (Two-sample Mann-Whitney U-test, p-value = 6.28x10^-18^); b Boxplots of the number of publications where co-medication pairs appeared co-mentioned (p-value = 1.52x10^-14^); c Boxplots of the odds ratio for dosage (p-value = 2.44x10^-19^). OR: Odds Ratio. (TIF) [file pdig.0000336.s007.tif]

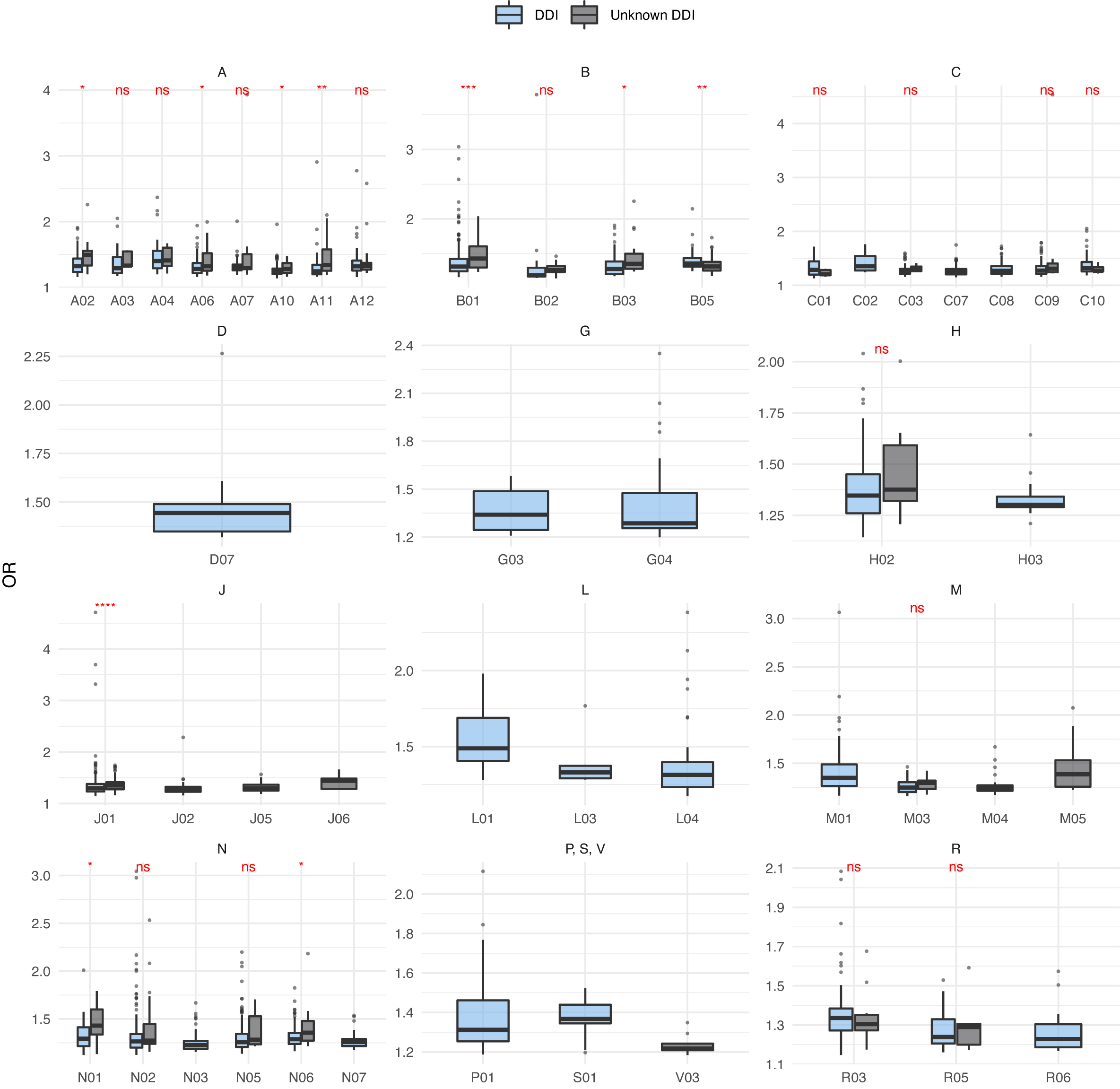

Supplement: S8 Fig — Boxplots indicating the OR and the DDI evidence among dosage adjusted co-medication pairs across the different index therapeutic drug groups. All significant differences showed small effect sizes, except vitamins (A11) with a moderate effect size. Two-sample Mann-Whitney U-test, where *: p-value ≤ 0.05, **: p-value ≤ 0.01, ***: p-value ≤ 0.001, ****: p-value ≤ 0.0001. Therapeutic groups with less than five observations for each DDI evidence class (DDI/Unknown DDI) are not shown. OR: Odds ratio. (TIF) [file pdig.0000336.s008.tif]

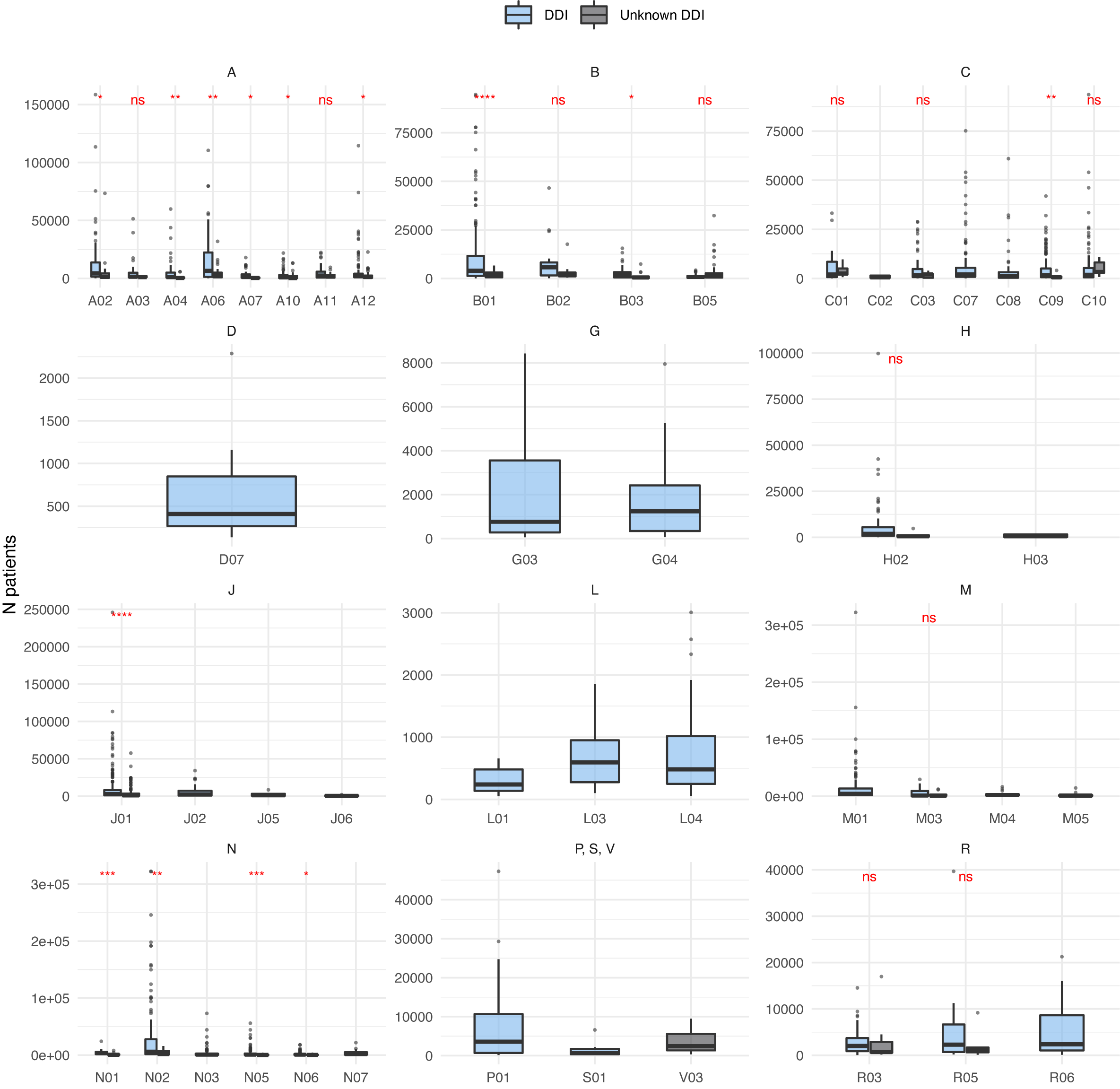

Supplement: S9 Fig — Boxplots indicating the prevalence (number of patients) and the DDI evidence among dosage adjusted co-medication pairs across the different index therapeutic drug groups. All significant differences showed small effect sizes, except anesthetics (N01) and antiemetics and antinauseants (A04) with a moderate effect size. Two-sample Mann-Whitney U-test, where *: p-value ≤ 0.05, **: p-value ≤ 0.01, ***: p-value ≤ 0.001, ****: p-value ≤ 0.0001. Therapeutic groups with less than five observations for each DDI class (DDI/Unknown DDI) are not shown. OR: Odds Ratio. (TIF) [file pdig.0000336.s009.tif]

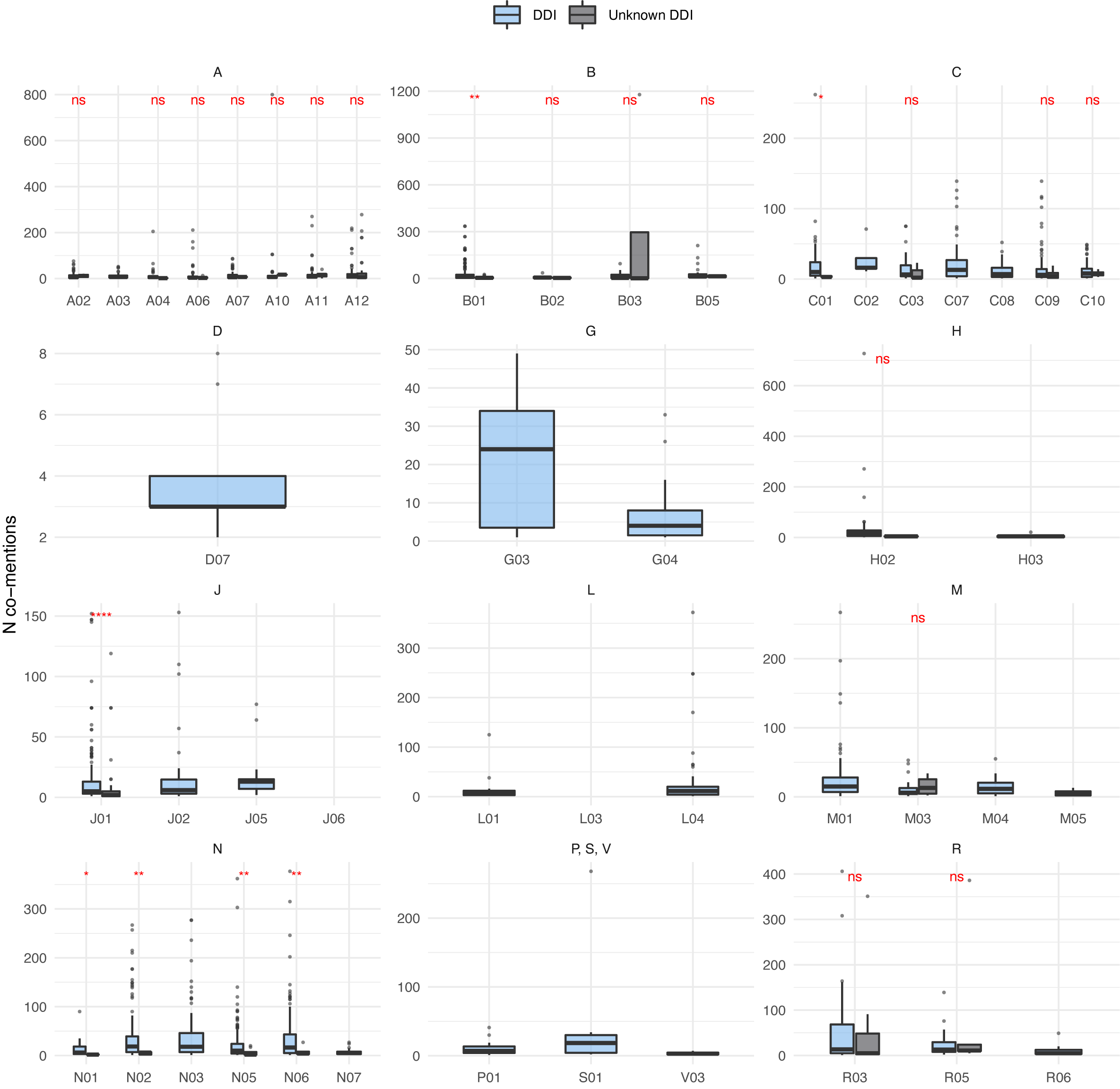

Supplement: S10 Fig — Boxplots indicating the number of publications with co-mentioning and the DDI evidence among dosage adjusted co-medication pairs across the different index therapeutic drug groups. All significant differences showed small effect sizes, except blood substitutes and perfusion solutions (B05), antibiotics (J01) and antiemetics and antinauseants (A04) with a large effect size; and mineral supplements (A12), vitamins (A11), drugs for functional gastrointestinal disorders (A03), analgesics (N02), antidiarrheals and antiiflammatory/antiinfective agents (A07), drugs for obstructive airway diseases (R03) and anesthetics (N01) with a moderate effect size. Two-sample Mann-Whitney U-test, where *: p-value ≤ 0.05, **: p-value ≤ 0.01, ***: p-value ≤ 0.001, ****: p-value ≤ 0.0001. Therapeutic groups with less than five observations for each DDI class (DDI/Unknown DDI) are not shown. OR: Odds Ratio. (TIF) [file pdig.0000336.s010.tif]

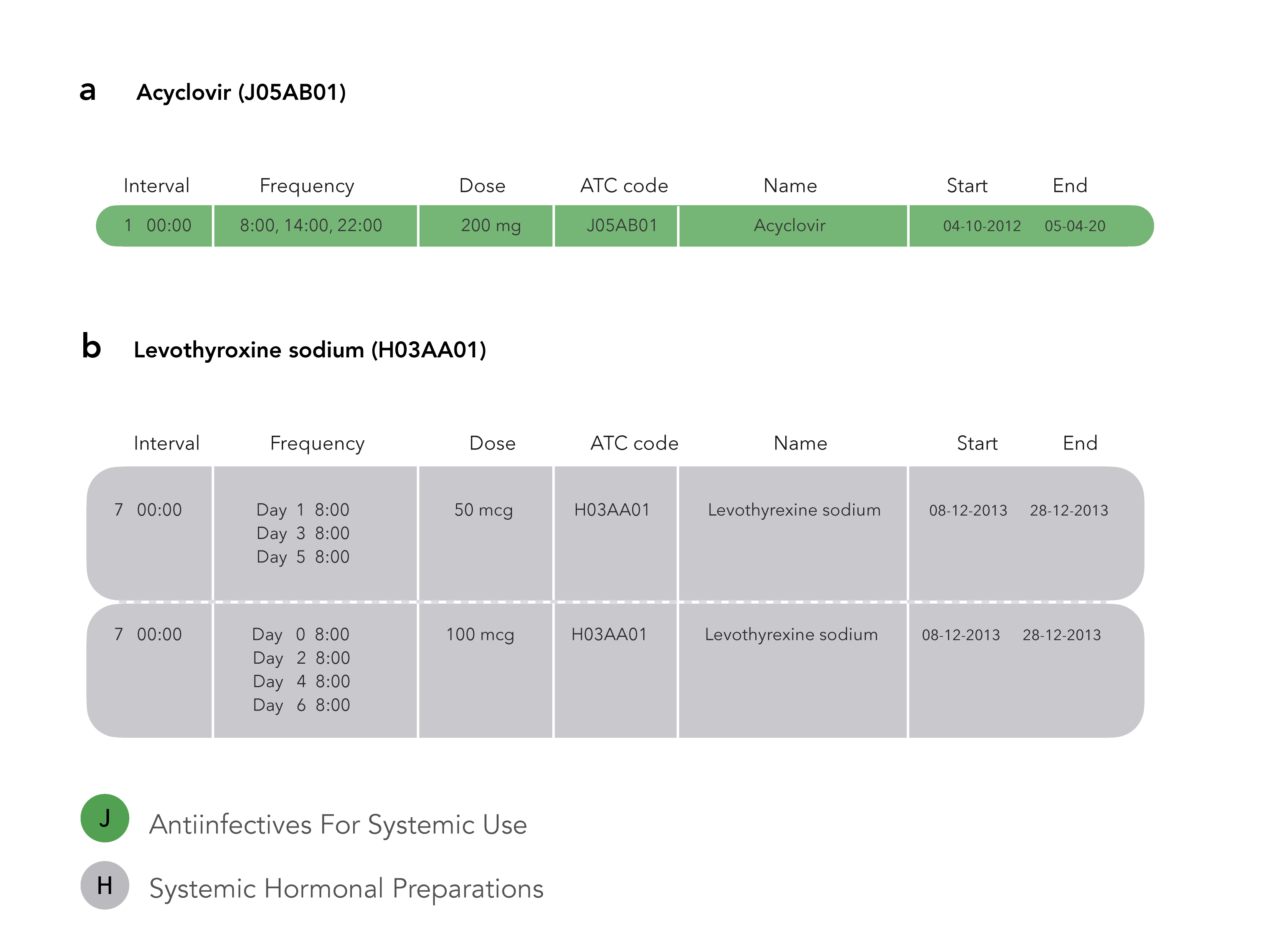

Supplement: S11 Fig — Calculation of prescribed dosage as the prescribed average daily dose (ADD). A prescription is a health-care program implemented by a physician or other qualified health care practitioner in the form of instructions that govern the plan of care for an individual patient. The term often refers to a health care provider’s written authorization for a patient to purchase a prescription drug from a pharmacist. Prescriptions can be classified into four different groups: (1) one-time prescriptions; (2) scheduled prescriptions; (3) Pro Necessitata (PN) or ‘as needed’ prescriptions; and (4) Variable dosage (VAO) prescriptions. One-time prescriptions consist on the administration of a single dose in a single day. As this type of prescriptions do not allow the possibility to study longitudinal dosage adjustments, we did not consider them for this study. PN consist of drugs only administered if needed and they are indicated with a maximum number of administrations/day that can be taken. An example of a PN prescription is the painkiller paracetamol or other analgesics indicated for the treatment of pain if the patient requires so after e.g. surgery. VAO drugs refer to drugs whose dosage is variable on a biochemical value or another physiological constant of the patient at the time of each administration. For example, this is the case for insulin, whose dosage depends on the blood glucose levels. For PN and VAO drug prescriptions, the ADD was coded categorically as ‘PN’ or ‘VAO’. Henceforth, quantitative ADD was calculated only for scheduled prescription types. Scheduled prescriptions consist of drug indications having an interval or cycle with a defined time pattern for each administration. Among this type, we can find many variations with different intervals and patterns. The figures a and b exemplify two different examples: a Dosing regimen for acyclovir (J05AB01), a drug used to treat infections caused by certain types of viruses (e.g. cold sores, shingles, chickenpox). The p [file pdig.0000336.s011.tif]

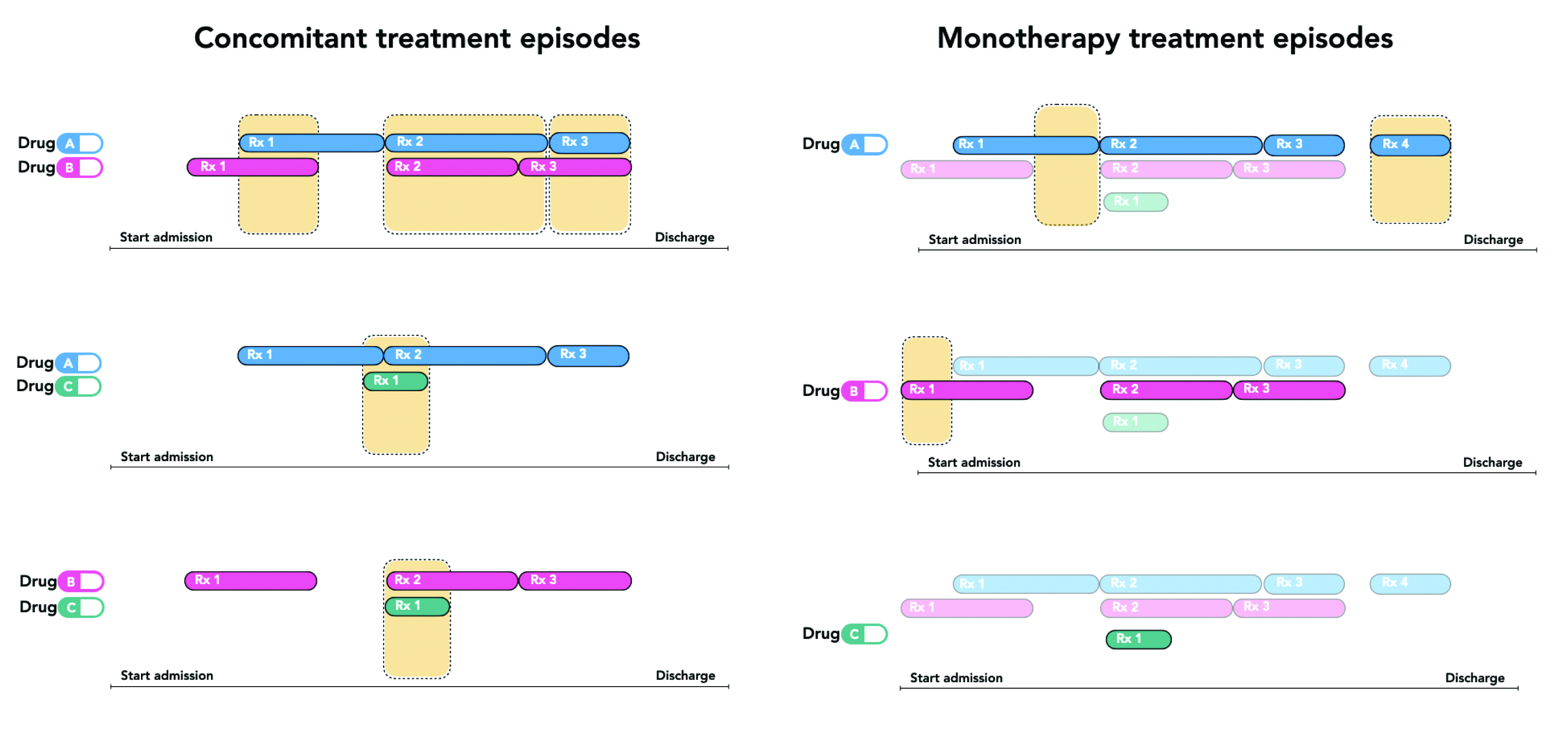

Supplement: S12 Fig — Patients can have a drug prescribed multiple times during an admission. We constructed concomitant treatment episodes as the intervals of time when two different drug prescriptions were contemporaneously active from start to end of the administration of a medication. Monotherapy treatment episodes were used as the reference treatment episodes as the intervals of time when a drug was not concomitantly given with any other drug. (TIF) [file pdig.0000336.s012.tif]

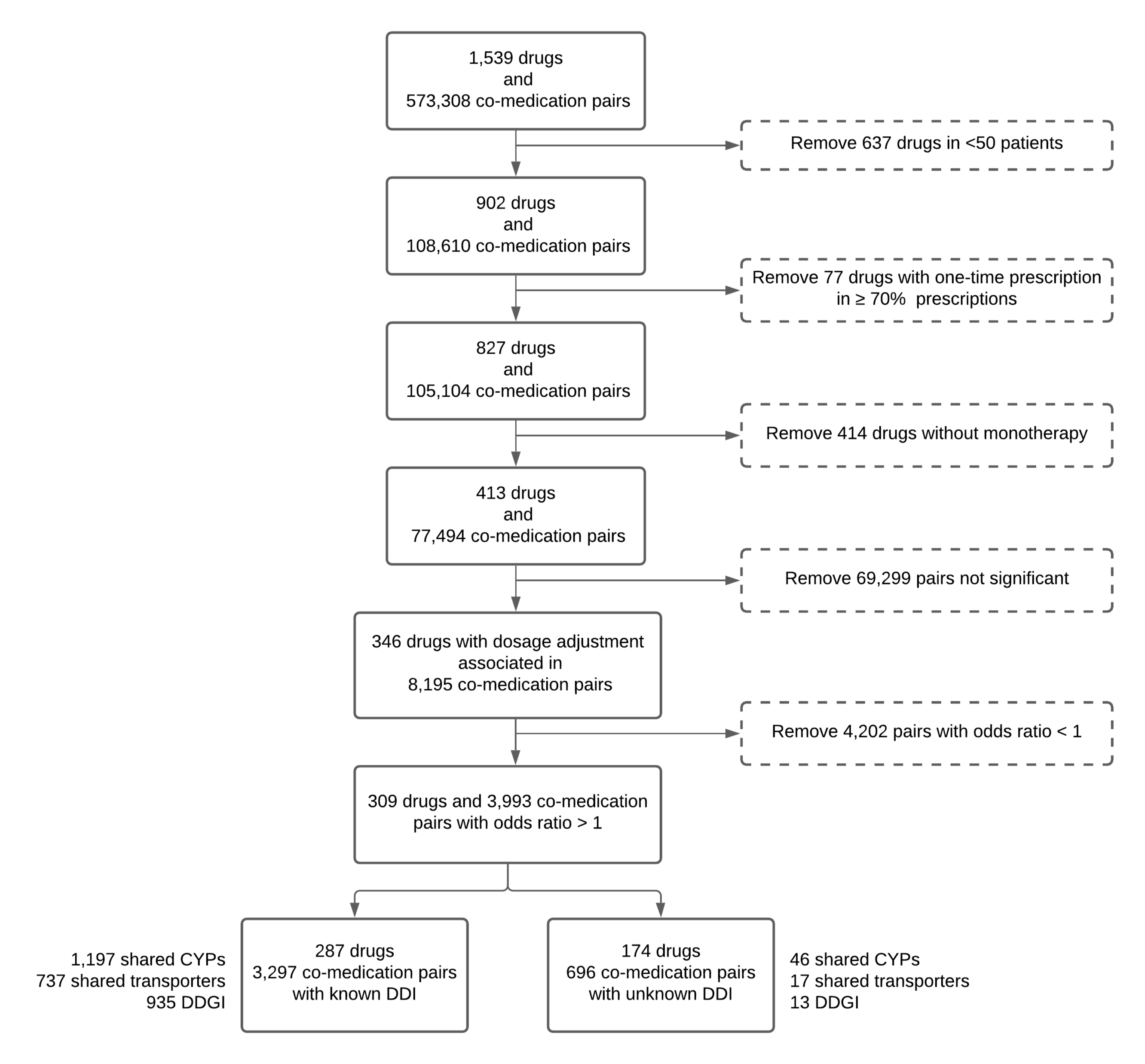

Supplement: S13 Fig — (TIF) [file pdig.0000336.s013.tif]

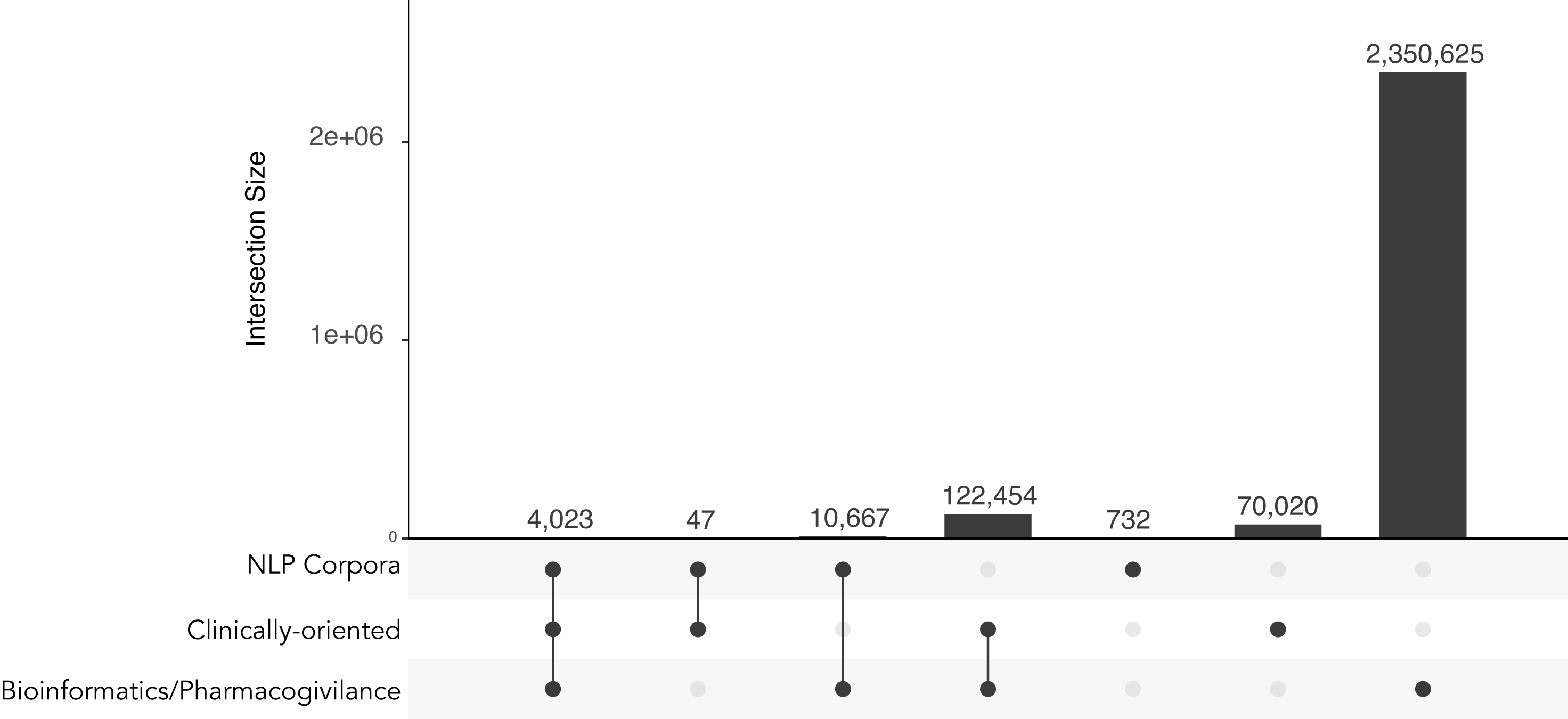

Supplement: S14 Fig — Upset plot showing the overlap of DDIs among different DDI source types: bioinformatics/pharmacovigilance, clinically oriented and Natural Language Processing (NLP) corpora. The upset plot is conceptually like a Venn diagram. The vertical histogram on the right shows the number of overlapping DDIs and the dots underneath show the source types containing them. The greatest overlap was of 122,454 DDIs between clinically oriented and bioinformatics/pharmacovigilance sources. DDI: drug-drug interaction. (TIF) [file pdig.0000336.s014.tif]

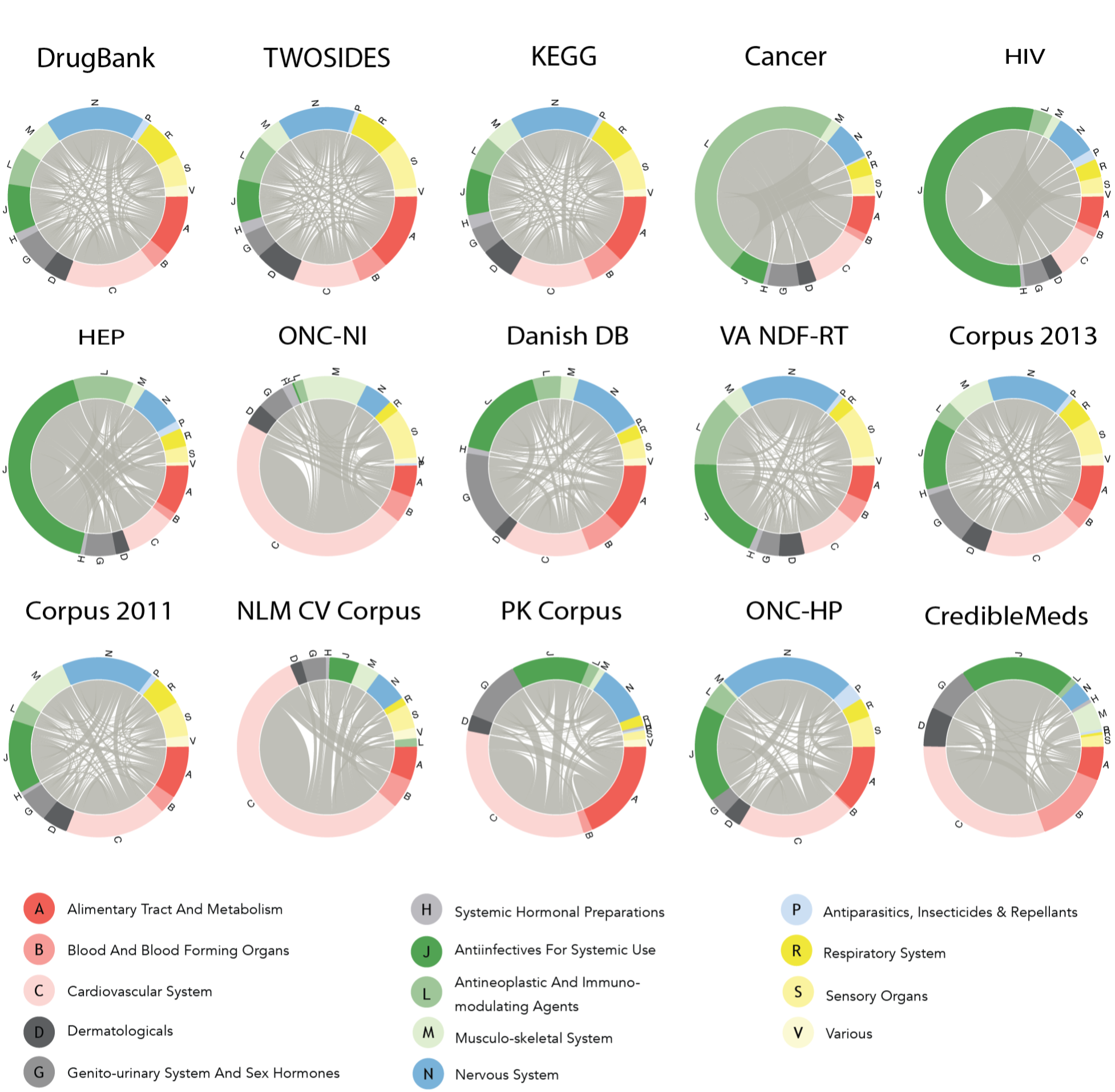

Supplement: S15 Fig — Proportion and relation between DDIs of different drug classes across the different databases used. Databases are sorted from left to right, top to bottom, according to the number of DDIs they contain (From DrugBank, the highest, to the lowest, CredibleMeds). Relation between drug classes is indicated by connecting bands, where the width is proportional to the number of pairs. The outer ring corresponds to the anatomical main group of the Anatomical Therapeutic Chemical (ATC) classification system. ONC-HP: ONC High Priority, ONC-NI: ONC Non-Interruptive, HEP: Hepatitis DDI database, HIV: Human Immunodeficiency Virus DDI database. (TIF) [file pdig.0000336.s015.tif]

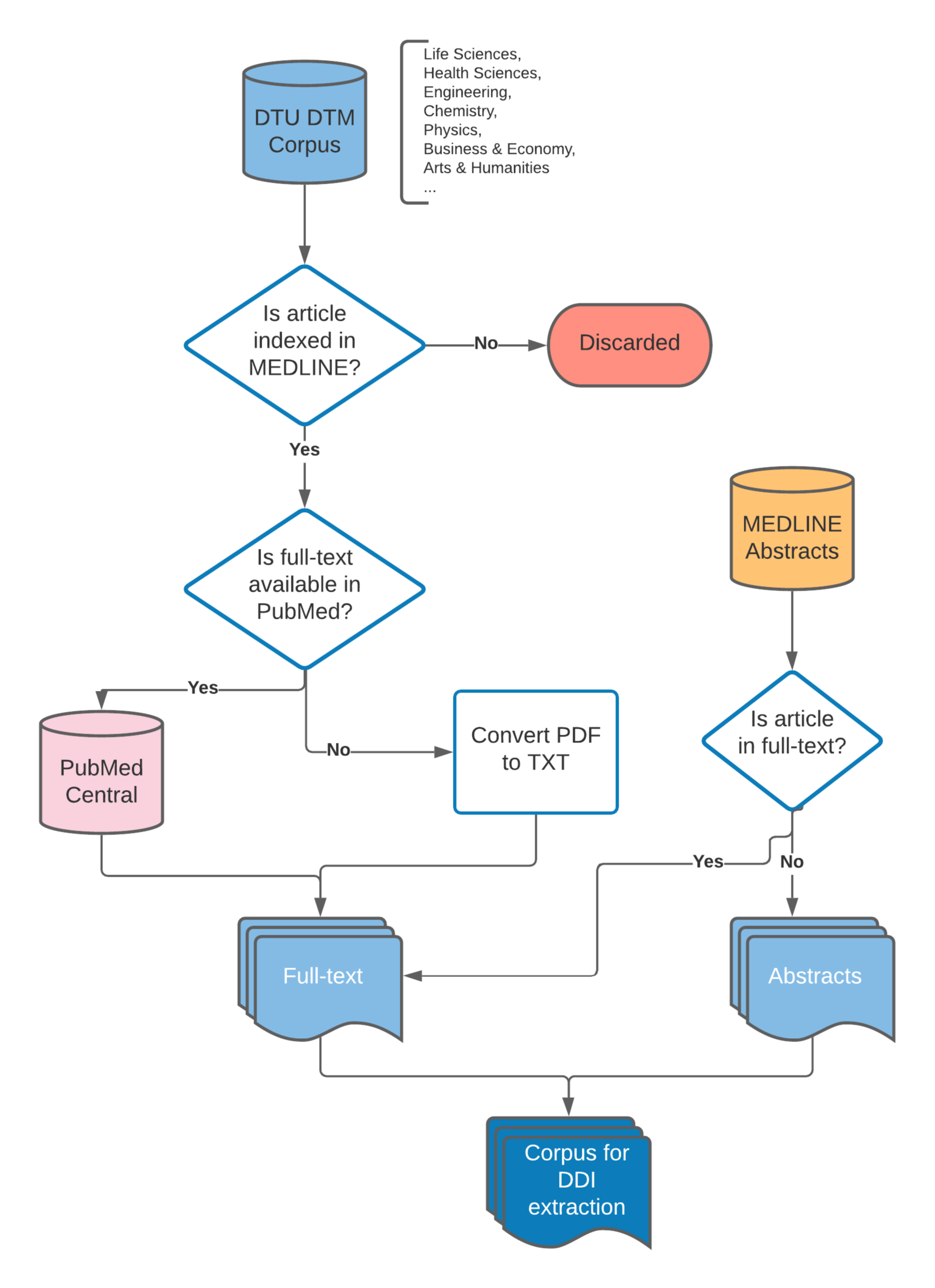

Supplement: S16 Fig — Diagram of the full-text article and abstract corpus generation for the text mining of DDIs. DTU DTM Corpus (~15 million articles), PubMed (~27 million articles) and MEDLINE were used for the collection of full-text articles and abstracts. (TIF) [file pdig.0000336.s016.tif]
