# Supplementary material for: Drug dosage modifications in 24 million in-patient prescriptions covering eight years: A Danish population-wide study of polypharmacy
Source: PLOS Digit Health. 2023 Sep 7;2(9):e0000336. doi: 10.1371/journal.pdig.0000336 (PMC10484442; doi:10.1371/journal.pdig.0000336)
Supplement: Attachment — Submitted filename: Leal et al. rebuttal response.pdf [file pdig.0000336.s028.pdf]

(PDIG-D-23-00017)

Dear Editor,

We would like to extend our gratitude to you, your team and the three reviewers for reading our manuscript carefully, and for providing valuable feedback and identifying aspects that could be revised. We genuinely believe that the manuscript with the general correction and clarification now has been significantly improved. Please, find below our responses to each of the comments from the reviewers and the editorial team. Review comments are set in black and our responses in blue immediately below.

With this response letter we return the revised manuscript along with updated additional files.

On behalf of the author group,

Søren Brunak

Reviewer-1

This study looked at an important topic and evaluated the drug dosage modifications in a chart reviews about polypharmacy. The study has been conducted in a methodologically sound way. However, given that there has only been a handful of retrospective and prospective studies on this topic. I think the main contribution of this study is to show the limited evidence on the polypharmacy with limitations about EHRs and their modifications. It will add much to literature.

We thank the reviewer for appreciating our study, including the methodology. We have now highlighted that the dosage information we analyse in the EHRs was in *structured form* stressing that this may give an advantage over e.g. questionnaires.

Major concerns

Polypharmacy concepts in western populations needs to be defined in a age specific definitions. It would have been better, if authors might have emphasized more on the introduction part about polypharmacy and stressed why this methodology was used. I am impressed by using Bayesian inferences and adjusted ORs given the complexity of drugs dosages.

We thank the reviewer for drawing our attention to the challenge of introducing the study better. As a response, we have now used “polypharmacy” more consistently in the Introduction. Regarding the request for a specific definition, we would like to emphasize that there is generally a call for a more uniform definition of polypharmacy in the

community (e.g., https://www.nature.com/articles/s43587-021-00045-3). That being so, we agree with the reviewer that the Introduction is not exactly clear on this point and have deleted the comment regarding increasing age and increasing polypharmacy. Importantly, we would also like to add that we systematically do adjust for age in all our analysis and in doing so, the developed method may answer previous problems regarding the limits stemming from simply counting the number of drugs when assessing polypharmacy.

Please include Clinical meaningful benefits of this study findings in discussion and conclusion sections, respectively.

We are grateful that the reviewer comments on this obviously missing aspect of our discussion. Aligned also with point 3 from this reviewer we have rewritten the Discussion and now included a subsection entitled "Clinical impact of co-medications pairs and DDIs" in it.

Reconsider and please rewrite the discussion with respect to your study findings.

Thank you! See comment above.

Reviewer-2

In this manuscript, the authors develop a model to characterize the complexity of polypharmacy and identify concomitant drug pairs subject to more frequent dosage adjustments. They also describe their association to outcomes, diagnoses, and laboratory values and classify co-medication pairs based on whether they have been labelled as DDIs previously and share pharmacokinetic properties. I do believe that the paper is both interesting and useful. I have no major concerns in regards to methodology, but do suggest that the authors should release their data and source code so that the readers can follow their work or check it.

We thank the reviewer for acknowledging the value of the work. Regarding the release of the data, we are not allowed to do that ourselves given the ethical permissions we have. These data are all EHRs from a large fraction of the Danish population in the period and they have not been deidentified and have been analysed in their raw form. We have just published another paper in PLOS Digital Health | https://doi.org/10.1371/journal.pdig.0000116 June 9, 2023, and the data in the present study are essentially the in-patient subset of that somewhat larger data set. In the recently published paper, we did not analyse the medication data. We have now inserted the same data availability statement outlining the application procedures. In the paper and supplementary information, we have only made available non-person-sensitive summary level data containing estimates for dosage adjustments and concomitant treatments. Access to the underlying person-sensitive data can be granted upon approval the Danish Data Protection Agency and the appropriate Danish Region following their procedures. This statement has now been updated. Regarding source code, we used Stan (v2.21), Python (v3.5.4), and R (v.3.4.0) for processing and subsequent statistical analyses and code can be made available upon request to the authors.

Reviewer-3

Firstly, congratulations to the authors on the successful submission of their paper for publication. The manuscript is well written and demonstrates a sound methodology.

A few general comments:
I suggest that the authors consider reorganizing the abstract to enhance its clarity and highlight the manuscript's contribution. It would be ideal to begin with the background and hypothesis, followed by a description of the methodology, and conclude with a brief summary of the results and their potential implications.

We have updated the abstract so the structure is more clear. It has also been shortened.

I recommend reorganizing the "Results" section for improved readability. Specifically, the first paragraph under "Condensation of millions of prescriptions into drug dosage patterns" could be more appropriately placed within the "Methods" section.

We thank the reviewer for this recommendation and agree that the readability of the “Results” section needed to be improved. In response we have, as suggested by the reviewer placed the subsection "Condensation of millions of prescriptions into drug dosage patterns" within the “Methods” section. Further, for improved readability we have renamed some of the subsections in the “Results” section (e.g., p. 4, line 92). Thus, the structure of the “Results” section is now: (1) In-hospital drug use and polypharmacy as a general and descriptive overview, (2) Co-medication pairs and likelihood of dosage adjustments, (3) Co-medication pairs in relation to clinical outcomes and multimorbidity, (4) Co-medication pairs in relation to DDIs, and (5) Co-medication pairs and their pharmacokinetic properties. Moreover, the “Discussion” section has been updated accordingly in response to point 3 raised also by Reviewer 1.

It would be beneficial to divide the "Discussion" section into sub-sections if feasible.

We have now included sub-sections in the “Discussion” section.

I suggest the authors consider increasing the font size of test in figures wherever possible.

Thank you, we have increased figures font size where possible.

Critical comments:
It is necessary for the authors to provide a justification for the use of a non-parametric test in their study. I recommend examining the variable distributions for normality such as the Kolmogorov-Smirnov (KS) test. Additionally, when presenting results in terms of p-values, I encourage the authors to report effect sizes such as Cliff's d or Cohen's d.

We assume the reviewer refers to the use of Two-sided Mann–Whitney U-test (or Wilcoxon rank-sum test) in the comparison of patient volumes, evidence and dosage adjustments between co-medication pairs with known/unknown DDI (p. 7, line 207). The choice was

made because this test is more robust to (1) outliers (compared to parametric tests based on means, such as the t-test) and (2) non-normality (i.e., response variable is skewed).

Patient volumes, literature evidence and dosage adjustments were all non-normally distributed ($P<0.05$, Shapiro-Wilk test of normality). Given the non-normality of the response variables, Cohen's d is not recommended. Instead, we used the R function 'wilcox_effsize' from the R package rstatix (version 0.7.2) that calculates effect size as Z statistics divided by the square root of the sample size. Significant differences in patient volumes, literature evidence and dosage adjustments between DDI and unknown DDI groups had a small effect size overall. When looking individually for each therapeutic group, we found that most significant differences had small effect size, but few with moderate and large effect sizes. This is now described in the paper.

We have updated the Methods section and corresponding data in results/figures by including the arguments presented above.

It would be ideal to provide a rationale for assuming a normal distribution for the specified set of priors in their study. It is also important to discuss the potential implications if a normal distribution assumption does not hold.

Historically, it has been preferable to use a conjugated prior, such as the gamma distribution. However, with recent advancements over the last decade, recommendations have changed. This is mostly due to the development of the Hamiltonian Monte Carlo No-U-Turn algorithm (Hoffman, 2014, https://jmlr.org/papers/volume15/hoffman14a/hoffman14a.pdf]) and evolution of the Stan programming language implementing this algorithm (https://mc-stan.org/). The normal prior for the standard deviation has some attractive properties in terms of convergence and further analysis, and our choice follows current recommendations. In our analyses, models were inspected for convergence with R-hat values below or equal to 1.1, tree depths not at maximal in any of the chains after warm-up, and zero divergences, indicating that posterior distributions reached the true parameter distribution. We think that these considerations are too basic to include in the manuscript.
